# Physician Workforce Diversity and Health Equity: It Is Time for Synergy in Missions!

**DOI:** 10.1089/heq.2019.0075

**Published:** 2019-11-21

**Authors:** Said A. Ibrahim

**Affiliations:** Department of Healthcare Policy and Research, Weill Cornell Medicine, New York, New York.

**Keywords:** diversity, healthcare disparities, health equity, underrepresented minority physician, workforce diversity, academic medicine, minority health

## Abstract

Diversity in academic medicine has regressed for the past two decades and the number of underrepresented minority trainees committed to academic careers in the biomedical sciences remains a significant challenge. Marked disparities in health care access and outcomes by race, gender, geography, and wealth remain, in part driven by social determinants of health. Despite widespread recognition that workforce diversity plays an important role in reducing disparities in health and health care, medical schools have not always linked their diversity efforts with their health equity efforts. The misalignment in academic diversity building and health equity initiatives represents a missed opportunity for mission synergy.

Despite near universal participation in the Association of American Medical Colleges (AAMC) Project 3000 by 2000 (Talamantes et al.^1^) to enroll 3000 underrepresented minorities (URMs) annually in U.S. medical schools by the year 2000, diversity in academic medicine has regressed for the past two decades. African Americans represent about 14% of the U.S. population, but only 4% of the physician workforce, and the number of African Americans enrolled in medical schools has dropped by 30% between 1997 and 2017.^1^ Hispanics and Latinos represent 17% of the U.S. population, but only 4% of medical doctors; native Americans represent about 2% of the U.S. population, but <0.4% of the physician workforce.^1^ In 2014, New York City, one of the most ethnically diverse communities in North America, URMs comprised only about 9% of the physician workforce. For a population that is 53% African American or Hispanic, only 12% of practicing physicians in New York City were URMs.^2^

The number of URM trainees committed to academic careers in the biomedical sciences also remains a significant challenge. URM scientists are less likely to compete successfully for National Institutes of Health funding to support their career development, relative to their white counterparts.^3^ Efforts to increase the diversity of the biomedical scientist workforce have not been effective for several reasons, including an inadequate pool of well-trained URM students at the pre- and postdoctoral levels, inconsistent funding for URM enrichment programs, and failure to reach URM scientists early in their careers to expose them to role models and mentors.

In an effort to address these challenges, many schools of medicine have established offices of diversity. Reporting directly to the dean of the school of medicine, diversity officers often serve as key members of a dean's executive leadership team. They are charged to develop and implement a comprehensive and strategic framework to attract and retain a diverse student body, faculty, and staff within the school of medicine. They also work across schools and departments to build structures and develop institutional policy designed to promote enterprise-wide diversity in all mission areas. They are accountable for continuous improvement through targeted data collection, analysis, and reporting on diversity initiatives. In other words, they serve as champions of diversity for the academic enterprise and strive to become trusted and inspirational leaders within their institutions.

The health care industry consumes about 18% of the nation's gross domestic product, yet U.S. health outcomes at every stage of the lifespan fall behind other developed countries. Marked disparities in health care access and outcomes by race, gender, geography, and wealth remain, in part driven by social determinants of health. Furthermore, demographic trends suggest by the year 2050, half of the U.S. population will be of a racial background other than white. At the population level, health care disparities in the United States cost an estimated $309 billion annually.^4^ The difference in life expectancy between the most privileged members of our society and those from disadvantaged backgrounds averages about 15 years.

Despite widespread recognition that workforce diversity can play an important role in reducing disparities in health and health care, medical schools have not always linked their diversity efforts with their health equity efforts. Rarely are institutional initiatives to address disparities centered within or even loosely associated with medical school offices of diversity. Academic leaders in the field of health equity are often career researchers and extramurally funded physician-scientists working within health care delivery systems. Most of the research to understand and intervene on health care disparities is conducted within the health care delivery systems and presented at national scientific meetings that predominantly attract audiences of investigators and clinician-educators, whose academic work is not always well aligned with medical school diversity initiatives. Many of the nation's research centers focused on health equity operate outside of the dean's offices of diversity. Similarly, many of the offices of diversity are led by accomplished educators and visionary administrators in medicine who may not be aware of the research and quality improvement efforts to address disparities within their affiliated health care delivery settings.

The misalignment in academic diversity building and health equity initiatives represents a missed opportunity for mission synergy. Leading health care organizations such as the National Academy of Medicine, the National Medical Association, AAMC, and the American Medical Association all recommend diversifying the health care profession as an important strategy to address disparities in health care. It has been known for some time now that compared with white physicians, URM physicians are more likely to practice medicine in URM communities where the need to expand access to care remains high. Lastly, a recent National Bureau of Economic Research Working Paper, “Does Diversity Matter for Health,” reported findings from a randomized controlled trial that suggested African American doctors, for instance, could help reduce cardiovascular mortality by 16 deaths per 100,000 per year. The investigators found that trust led to increased preventive care—leading to a 19% reduction in cardiovascular mortality gap between African American and white males.^5^ Whether the racial/ethnic concordance of patients and providers is a source of improved outcomes for minority patients has been a subject of research and reviews.^6^

The national mandates to achieve representative diversity in the physician workforce and health equity share a common moral underpinning. The link between the two is well recognized. It is past time for medical school deans to seek ways to synergize their diversity initiatives with their health equity efforts and vice versa. Synergy entails the process of combining skill sets, investments/resources, and perspectives, but requires collaboration, which in turn requires solid relationships and structures.^7^ Bringing leaders in diversity together with leaders in health equity will reveal untapped opportunities and help deans direct institutional resources to more effectively advance workforce diversity and health equity. This viewpoint recognizes the complexity and the multifactorial nature of health equity. It is also not a call to dilute or undermine ongoing national efforts on either medical workforce diversity or health equity. But rather to envision strategies that make the sum of these efforts greater than their individual components.

## Abbreviations Used

AAMC: Association of American Medical Colleges
URMs: underrepresented minorities
